# A comparative study of machine learning methods for time-to-event survival data for radiomics risk modelling

**DOI:** 10.1038/s41598-017-13448-3

**Published:** 2017-10-16

**Authors:** Stefan Leger, Alex Zwanenburg, Karoline Pilz, Fabian Lohaus, Annett Linge, Klaus Zöphel, Jörg Kotzerke, Andreas Schreiber, Inge Tinhofer, Volker Budach, Ali Sak, Martin Stuschke, Panagiotis Balermpas, Claus Rödel, Ute Ganswindt, Claus Belka, Steffi Pigorsch, Stephanie E. Combs, David Mönnich, Daniel Zips, Mechthild Krause, Michael Baumann, Esther G. C. Troost, Steffen Löck, Christian Richter

**Affiliations:** 1 OncoRay - National Center for Radiation Research in Oncology, Faculty of Medicine and University Hospital Carl Gustav Carus, Technische Universität Dresden, Helmholtz-Zentrum Dresden – Rossendorf, Dresden, Germany; 2 German Cancer Research Center (DKFZ), Heidelberg and German Cancer Consortium (DKTK) partner site Dresden, Dresden, Germany; 3 German Cancer Research Center (DKFZ), Heidelberg and German Cancer Consortium (DKTK) partner site Berlin, Berlin, Germany; 4 German Cancer Research Center (DKFZ), Heidelberg and German Cancer Consortium (DKTK) partner site Essen, Essen, Germany; 5 German Cancer Research Center (DKFZ), Heidelberg and German Cancer Consortium (DKTK) partner site Frankfurt, Frankfurt, Germany; 6 German Cancer Research Center (DKFZ), Heidelberg and German Cancer Consortium (DKTK) partner site Munich, Munich, Germany; 7 German Cancer Research Center (DKFZ), Heidelberg and German Cancer Consortium (DKTK) partner site Tübingen, Tübingen, Germany; 8 National Center for Tumor Diseases (NCT), partner site Dresden, Dresden, Germany; 9 German Cancer Research Center (DKFZ), Heidelberg, Germany; 10 Department of Radiotherapy and Radiation Oncology, Faculty of Medicine and University Hospital Carl Gustav Carus, Technische Universität Dresden, Dresden, Germany; 11 Helmholtz-Zentrum Dresden – Rossendorf, Institute of Radiooncology - OncoRay, Dresden, Germany; 12 Department of Nuclear Medicine, Faculty of Medicine and University Hospital Carl Gustav Carus, Technische Universität Dresden, Dresden, Germany; 13 Helmholtz-Zentrum Dresden-Rossendorf, PET Center, Institute of Radiopharmaceutical Cancer Research, Dresden, Germany; 14 Clinic of Radiation Oncology, Teaching Hospital Dresden - Friedrichstadt, Technische Universität Dresden, Dresden, Germany; 15 Department of Radiooncology and Radiotherapy, Charité University Hospital, Berlin, Germany; 16 Department of Radiotherapy, Medical Faculty, University of Duisburg-Essen, Essen, Germany; 17 Department of Radiotherapy and Oncology, Goethe-University Frankfurt, Frankfurt, Germany; 18 Heidelberg Ion Therapy Center (HIT), Department of Radiation Oncology, University of Heidelberg Medical School, Heidelberg, Germany; 19 Department of Radiation Oncology, Ludwig-Maximilians-Universität, Munich, Germany; 20 Clinical Cooperation Group, Personalized Radiotherapy in Head and Neck Cancer, Helmholtz Zentrum, Munich, Germany; 21 Department of Radiation Oncology, Technische Universität München, München, Germany; 22 Institute of Innovative Radiotherapy (iRT), Helmholtz Zentrum München, Oberschleißheim, Germany; 23 Department of Radiation Oncology, Faculty of Medicine and University Hospital Tübingen, Eberhard Karls Universität Tübingen, Tübingen, Germany

## Abstract

Radiomics applies machine learning algorithms to quantitative imaging data to characterise the tumour phenotype and predict clinical outcome. For the development of radiomics risk models, a variety of different algorithms is available and it is not clear which one gives optimal results. Therefore, we assessed the performance of 11 machine learning algorithms combined with 12 feature selection methods by the concordance index (C-Index), to predict loco-regional tumour control (LRC) and overall survival for patients with head and neck squamous cell carcinoma. The considered algorithms are able to deal with continuous time-to-event survival data. Feature selection and model building were performed on a multicentre cohort (213 patients) and validated using an independent cohort (80 patients). We found several combinations of machine learning algorithms and feature selection methods which achieve similar results, *e*.*g*., MSR-RF: C-Index = 0.71 and BT-COX: C-Index = 0.70 in combination with Spearman feature selection. Using the best performing models, patients were stratified into groups of low and high risk of recurrence. Significant differences in LRC were obtained between both groups on the validation cohort. Based on the presented analysis, we identified a subset of algorithms which should be considered in future radiomics studies to develop stable and clinically relevant predictive models for time-to-event endpoints.

## Introduction

In the era of patient specific cancer therapy, radiomics is a new and promising field in radiation oncology^1^. Radiomics aims to predict patient specific outcomes based on high-throughput analysis and mining of advanced imaging biomarkers by machine learning algorithms. It has shown promising results in several studies on lung, head and neck, breast as well as brain tumours^2–7^. In radiomics, feature selection is used to identify prognostic biomarkers (signature) and to reduce the dimensionality of the feature space^8^. Machine learning algorithms subsequently use the signature to construct predictive models by learning the decision boundaries of the underlying data distribution. A variety of feature selection methods and machine learning approaches exist. However, most radiomics studies only consider the combination of one feature selection with one learning algorithm. For instance, L. van Dijk *et al*.^9^ used the Pearson correlation coefficient to identify relevant image features in combination with the Lasso regularisation to develop a multivariable logistic regression model. In contrast, Kickingereder *et al*.^3^, used a supervised principal component analysis based on coefficients of the Cox regression model to develop the radiomics signature in combination with a multivariate Cox regression model for prediction of survival. To date it is not clear whether these methodological choices led to models with the highest prognostic accuracy.

Therefore, a systemic evaluation to identify a set of suitable feature selection methods and learning algorithms is a critical step to develop clinically relevant radiomics risk models. Thus far, only few studies have performed such an evaluation. Recently, Parmar *et al*.^10,11^, investigated different algorithms in two different studies for patients with non-small cell lung (NSCLC) cancer and locally advanced head and neck squamous cell carcinoma (HNSCC). However, in these studies the outcome of interest, overall survival (OS), was transformed to a binary endpoint. While dichotomisation of the endpoint is a method for stratifying patient groups, it incurs the risk of biasing prediction accuracy^12^. Therefore we avoid dichotomisation of continuous time-to-event data, and instead base patient stratification on the predicted risk.

In the present study we systematically assessed 11 machine learning algorithms and 12 feature selection methods for the prediction of continuous time-to-event data. Pre-treatment computed tomography (CT) scans were recorded in 293 HNSCC patients from a multicentre cohort. The patients were divided into strictly separated exploratory (n = 213) and validation (n = 80) cohorts. We then used the CT images of the exploratory cohort to build risk models for loco-regional tumour control (LRC) and overall survival (OS). Subsequently, we assessed both, predictive performance of the models and patient risk stratification, for each combination of feature selection and learning algorithm on the validation cohort. Furthermore we assessed the robustness of the selected signatures for each feature selection method using the intra-class correlation coefficient (ICC)^12^ calculated for rotated and translated images of the exploratory cohort. In addition we evaluated the predictive performance of our models to the radiomics signature previously defined by Aerts *et al*.^5^. The evaluations above led to the identification of a subset of useful feature selection and learning algorithms for time-to-event survival data.

## Material and Methods

### Patient cohorts

In this study, two cohorts with a total of 293 patients from different institutions were included. All patients suffered from histologically confirmed loco-regionally advanced HNSCC and received primary radiochemotherapy. Patients were allocated to an exploratory and validation cohort with a ratio of 2:1 based on the different included studies rather than on the treatment places. The exploratory cohort included 213 patients. 152 of the 213 patients were treated in one of the seven partner sites of the German Cancer Consortium Radiation Oncology Group (DKTK-ROG)^13^ between 2005 and 2011. The remaining 61 patients of the exploratory cohort were treated at the University Hospital Dresden (UKD, Germany) between 1999 and 2006. The validation cohort consisted of 80 patients. 50 of the 80 patients were treated within a prospective clinical trial [NCT00180180, ref.^14^] at the UKD between 2006 and 2012. The remaining 30 patients were treated at the UKD and the Radiotherapy Center Dresden-Friedrichstadt (RCDF) between 2005 and 2009. The clinical characteristics of both cohorts are summarised in Supplement A. Ethical approval for the multicentre retrospective analyses of clinical and imaging data was obtained from the Ethics Committee at the Technische Universität Dresden, Germany, EK177042017. All analyses were carried out in accordance with the relevant guidelines and regulations. Informed consent was obtained from all patients.

### Image pre-processing and feature extraction

**Figure 1:**
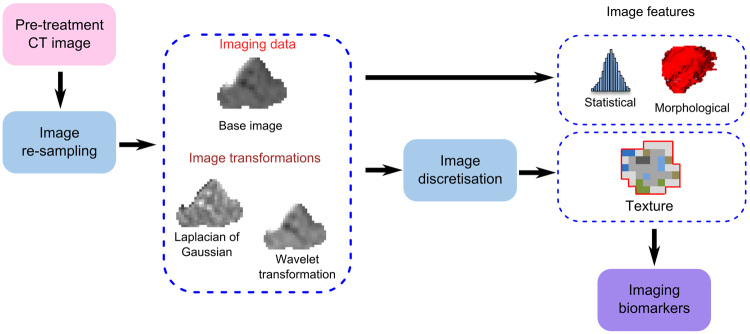
Illustration of image pre-processing and feature extraction.

Figure 1 illustrates the image pre-processing and feature extraction workflow. Prior to analysing the pre-treatment computed tomography (CT) scans without contrast agent, the gross tumour volume (GTV) was manually delineated taking into account patient examination and the findings of additional imaging modalities. Voxels in each CT image volume were re-sampled to an isotropic voxel size of 1.0 × 1.0 × 1.0 mm^3^ to correct for different voxel spacings and slice thicknesses between different centres^5^. Subsequently, the GTV was re-segmented to cover only soft tissue voxels between −150 and 180 Hounsfield units, removing voxels containing air and bone. Spatial filtering was applied to the base image to quantify additional image characteristics such as edges and blobs. We performed a stationary coiflet-1 wavelet transformation along the three spatial dimensions which produced eight transformed images in addition to the base image. A mean Laplacian of Gaussian (LoG) image of five different kernel widths (0.5 mm, 1.0 mm, 2.0 mm 3.0 mm, 5.0 mm, respectively, ref.^15^) was generated from the base CT image as a further image volume.

In each image set 18 statistical, 18 morphological, 30 histogram-based and 95 texture features were extracted from the GTV, leading to 1610 features in total. The following texture matrices were used: grey-level co-occurrence (GLCM)^16^, grey-level run length (GRLM)^17,18^, neighbourhood grey tone difference (NGTDM)^19^, grey-level size zone (GLZSM)^20^, grey-level distance zone (GLDZM)^21^ and neighbourhood grey level dependence (NGLDM)^22^ matrix. All features were calculated using a volumetric approach, and not by slice. Mathematical descriptions of all features are published in ref.^23^. The GTV was discretised using 64 quantization levels before calculation of texture matrices and the intensity histogram^24,25^. GLCM and GLRLM-based features were first calculated for each of the 13 different spatial directions and subsequently averaged. All features were normalised on the exploratory cohort using z-score normalisation. The resulting scale and shift constants were applied to the independent validation cohort. For image pre-processing and feature extraction we developed in-house software based on Python 2.7 (Python Software Foundation).

### Feature selection methods and machine learning algorithms

In the present study different feature selection methods were considered: (I) correlation-based methods: Pearson, Spearman; (II) feature selection algorithms based on mutual information optimisation: mutual information maximisation (MIM), mutual information feature selection (MIFS), minimum redundancy maximum relevance (MRMR); and (III) model-based approaches: a univariate (uni)- and a multivariate (multi)-Cox-regression model, a random forest minimal depth (RF-MD), a random forest variable importance (RF-VI), a random forest based on maximally selected rank statistics variable importance (MSR-RFVI) and a random forest based on permutation variable importance (PVI-RF). Additionally, we selected features at random (RAND) and performed no feature selection (None).

The comparison of different machine learning algorithms included the following non-parametric models: (I) the Cox model, the NET-Cox method with lasso and elastic-net regularisation; (II) models based on boosting trees (BT): BT-Cox, BT-CIndex; (III) boosting gradient linear models (BGLM): BGLM-Cox, BGLM-CIndex; and (IV) random forest based methods: random survival forest (RSF), random forest using maximally selected rank statistics (MSR-RF). Furthermore we investigated the following full-parametric models (V): survival regression (Survival-Regression) and models based on the Weibull distribution: BT- and BGLM-Weibull. A short description of all of these methods can be found in Supplement B. All feature selection methods and machine learning algorithms assessed here are able to handle continuous time-to-event data.

### Radiomics modelling framework

**Figure 2:**
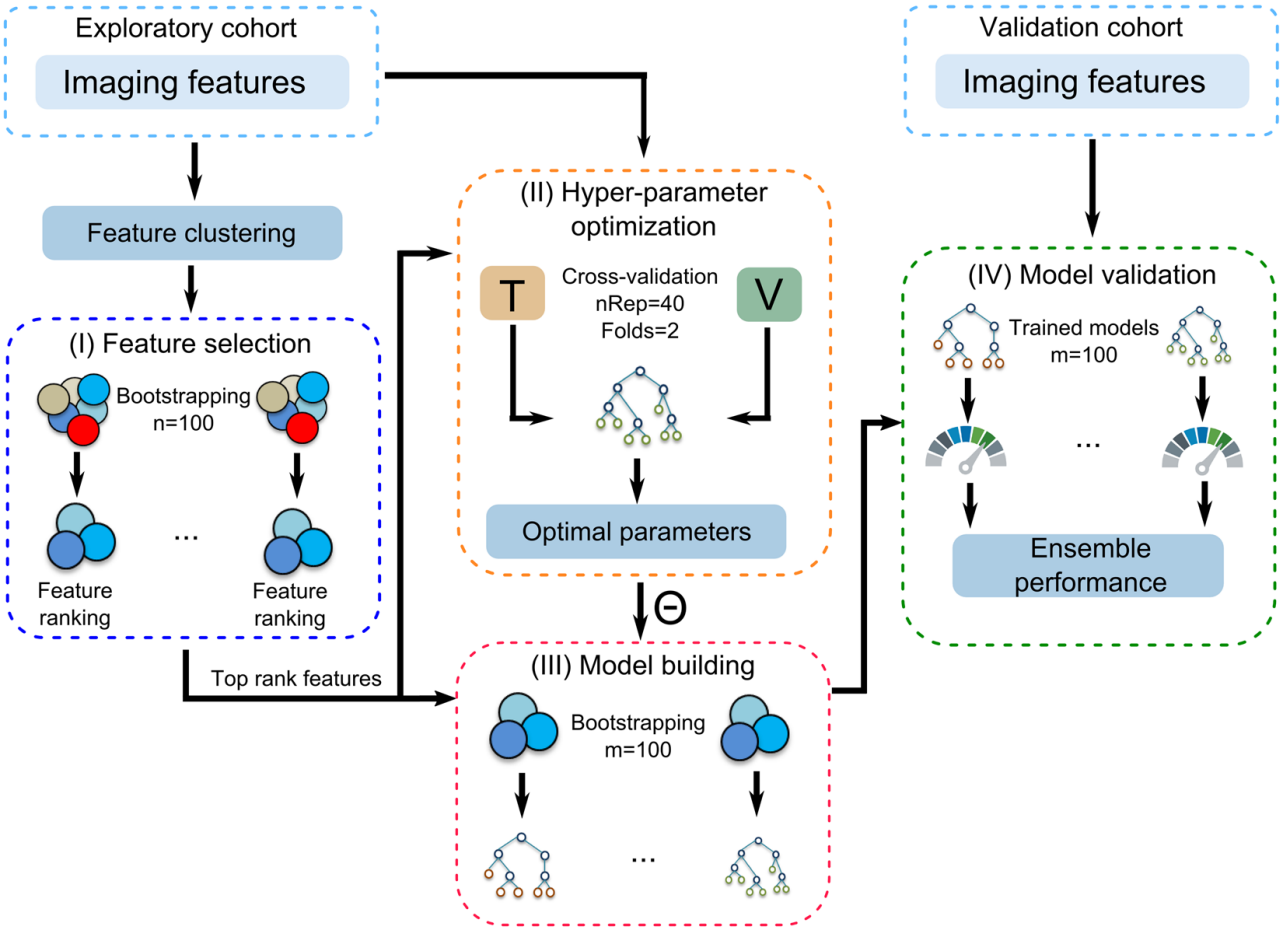
Illustration of the major radiomics processing chain within the radiomics modelling framework (RMF). (I) feature clustering and selection to identify prognostic biomarkers, (II) automatic hyper-parameter optimisation Θ for each model using a 2-fold cross validation with 40 times repetitions based on the exploratory cohort, (III) model building and (IV) model validation were performed.

A radiomics modelling framework (RMF) was developed to create radiomics signatures, to optimise the hyper-parameters of machine learning algorithms, to train predictive models, and subsequently determine the predictive performance of the models as well as to perform the Kaplan-Meier survival analyses on validation data. Figure 2 shows the RMF and its four major processing steps: (I) feature selection, (II) hyper-parameter selection, (III) model building and (IV) model validation. The RMF was developed in-house using R 3.3.2^26^.

### Feature selection

After feature extraction, feature clustering was performed on the exploratory cohort to obtain an initial non-redundant set of biomarkers^27^. Highly correlated imaging biomarkers (Spearman correlation coefficient (SCC) >0.90) were clustered using hierarchical clustering^28^. The resulting clusters were represented by a meta-feature calculated by averaging over all features within the cluster. Negatively correlated features were inverted before averaging. A total of 229 non-singular clusters were created. The same clusters and meta-features were also generated for the validation cohort.

After clustering, the feature set of the exploratory cohort was used to identify the most relevant features using feature selection algorithms. Feature selection was repeated *n* = 100 times using *n* bootstrap samples (*i*.*e*., 0.632 bootstrap method with replacement) of the exploratory cohort to ensure the selection of stable features. Feature selection ranks each feature according to a score, which depends on the method used. The top 20 best ranking features were selected from each bootstrap sample. We subsequently aggregated the selected features *j* over the bootstraps by calculating an importance score *I*_*j*_, defined as

1 $${I}_{j}=\frac{{\sum }_{i=1}^{n}\sqrt{{R}_{ij}}}{oc{c}_{j}^{2}},$$

where *R*_*ij*_ defines the rank within the *i*-th bootstrap sample and *occ*_*j*_ the frequency of occurrence of feature *j* over all bootstrap samples. The feature rank aggregation score is based on the enhanced Borda score^29^, with the difference being that feature occurrence receives a greater weight.

### Hyper-parameter optimisation

After feature selection and rank aggregation, hyper-parameters of the machine learning algorithms, such as signature size or algorithm-specific settings were optimised for each combination of feature selection and machine learning algorithm. A major objective of hyper-parameter tuning is to limit model overfitting. Overfitting would otherwise lead to poor predictive performance on unseen data. The individual hyper-parameter set Ω of each learning algorithm *A* was tuned by using an internal 2-fold cross validation scheme which was repeated 40 times (*n*_Rep_ = 40) based on the exploratory cohort.

Hyper-parameter optimisation was performed using a grid search through a pre-defined hyper-parameter space. The objective of the hyper-parameter optimisation is to minimise a loss function *L*(*Y*, *X*, *A*) over the internal training and validation folds, *X* and *Y*, respectively, by a trained learning algorithm *A* to obtain an optimal set of parameters Ω*:

2 $$\,{{\rm{\Omega }}}^{\ast }=\mathop{{\rm{argmin}}}\limits_{\gamma \in {\rm{\Omega }}}\,[L({Y}^{{\rm{valid}}},{X}^{{\rm{train}}},{A}_{\gamma }({X}^{{\rm{train}}}))],$$

3 $$\,{\rm{L}}(Y,X,A)={\gamma }_{1}+{\gamma }_{2}+{\gamma }_{3}$$

with

4 $$\,{\gamma }_{1}=\,{\rm{\max }}(\frac{1-\alpha }{{({\mu }_{{\rm{train}}}-\alpha )}^{2}}-1,\frac{1-\alpha }{{({\mu }_{{\rm{valid}}}-\alpha )}^{2}}-1),$$

5 $${{\rm{\gamma }}}_{2}=50(\frac{1}{{(1-{\mu }_{{\rm{bal}}})}^{{\rm{2}}}}-1),$$

6 $$\,{\gamma }_{3}=\{\begin{array}{cccc}1000 & {{\rm{\mu }}}_{{\rm{train}}} > {\rm{\alpha }}\cup {{\rm{\mu }}}_{{\rm{valid}}} < {\rm{\alpha }} & {\rm{or}} & {{\rm{\mu }}}_{{\rm{train}}} < {\rm{\alpha }}\cup {{\rm{\mu }}}_{{\rm{valid}}} > {\rm{\alpha }}\\ 0 &  & {\rm{otherwise}} & \end{array}\}.$$

Here, *μ*_train_ and *μ*_valid_ are the average prediction accuracy of the internal training and validation folds and *μ*_bal_ is the difference in average prediction accuracy between training and validation folds. The correction factor *α* = 0.5 represents the performance of a random experiment. The terms *γ*_1_, *γ*_2_ define penalties for the training-test error to minimise both test error and differences between training and test error. This leads to hyper-parameter sets where train and test error are more similar (balanced), which may increase model generalisability. The penalty term *γ*_3_ accounts for the discordance in train and test errors, to avoid selecting hyper-parameter sets where the predictions on the training set were concordant with the outcome, yet discordant on the test set.

### Model building and validation

Model training was performed *m* = 100 times using bootstrap samples (*i*.*e*., 0.632 bootstrap method with replacement) of the exploratory cohort for each combination of feature selection method and machine learning algorithm. The learning algorithms were trained on the generated bootstrap samples based on the top rank features as well as the optimised hyper-parameter set. Afterwards, an ensemble prediction^30^ was made by averaging the predicted risk scores for each model using data of the independent validation cohort. The ensemble model performance on the validation cohort was assessed using the concordance index (C-Index)^31,32^. The C-Index is a generalisation of the area under the curve for continuous time-to-event survival data. C-Index = 0.5 describes a random prediction whereas a perfectly predicting model has C-Index = 1.0.

### Clinical endpoints and statistical analysis

The clinical endpoints LRC and OS were calculated from the first day of radiochemotherapy to the date of event or censoring. The number of events for LRC and OS were 86 and 120 for the exploratory cohort, and 26 and 51 for the validation cohort, respectively.

In the present study, four analyses were performed: (I) the predictive performance of all combinations of feature selection methods and machine learning algorithms was evaluated based on the validation C-Index of the ensemble of models. (II) The median and standard deviation of the validation C-Indices of a feature selection method over all machine learning algorithms and *vice versa* was assessed to measure the variance induced by the respective algorithms. (III) The robustness of the radiomic signatures was assessed by applying different image rotations (±2°, ±6°, ±10°) and translations in x-y-direction (0.25 mm, 0.75 mm) for all combinations to the exploratory cohort and subsequently calculating the ICC for each feature selected by the various feature selection methods. (IV) Patients were stratified into a low and high risk group based on the predicted risk of the radiomics models. The cut-off value used for stratification was selected by using 1000 bootstrap samples based on the exploratory cohort. The fraction of significant stratification results (power) was calculated for each cut-off, leading to the optimal value which has the largest power^33^. Cut-off values were applied to the validation cohort unchanged. Survival curves were estimated by the Kaplan-Meier method and compared by log-rank tests. Finally, (V) validation performance for both endpoints was determined for the external signature, obtained by Aerts *et al*.^5^. In addition, we performed a statistical test to compare the C-Indices of the best performing models for LRC using the R package “compareC”^34^. Two-sided tests were applied and *p*-values < 0.05 were considered as statistically significant.

## Results

### Prognostic performance of feature selection and machine learning methods

**Figure 3:**
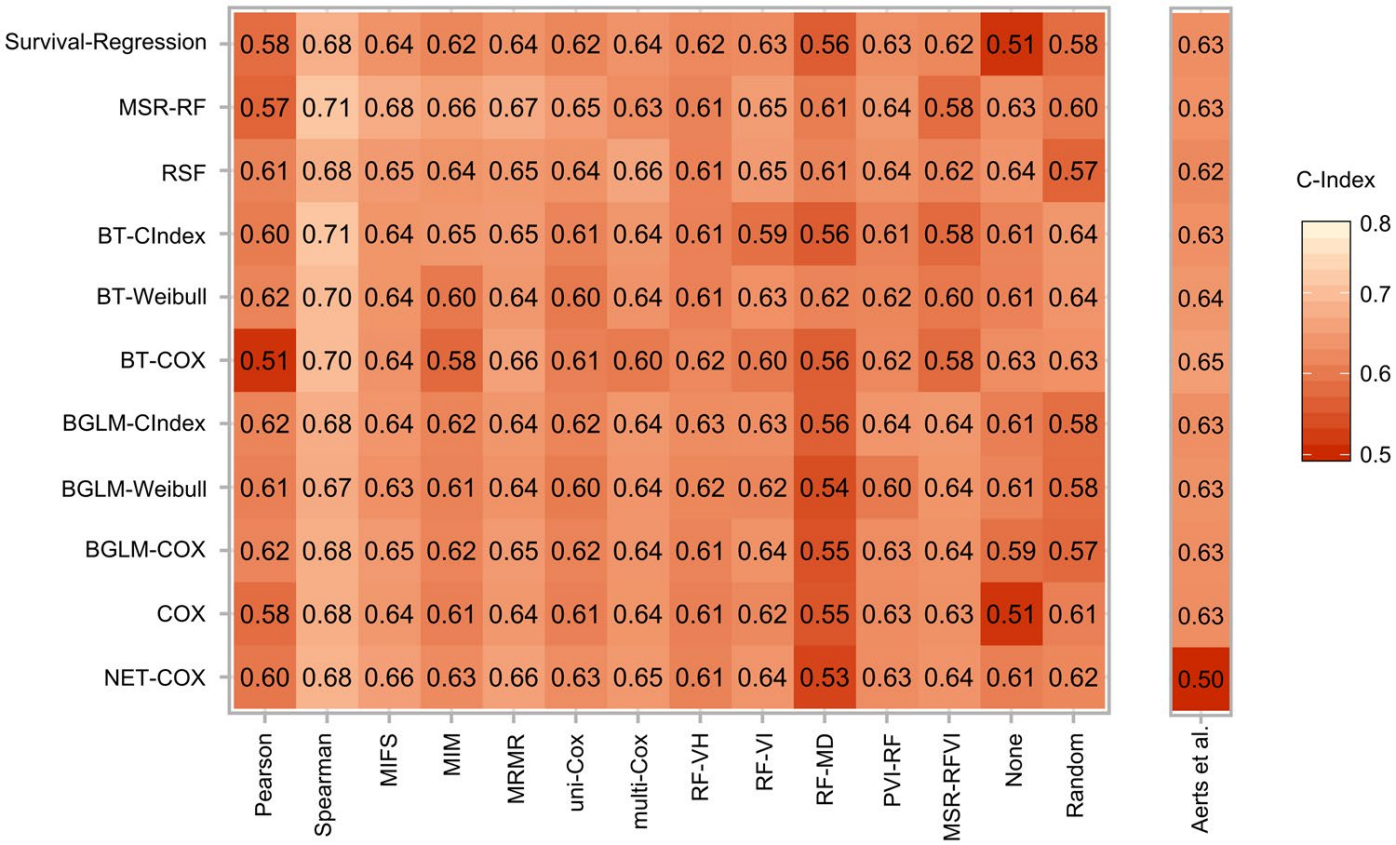
Heatmap depicting the concordance indices depending on the feature selection method (rows) and learning algorithm (columns) for the validation cohort as well as the Aerts *et al*.^5^ signature for loco-regional tumour control.

The prognostic performance of different feature selection methods combined with machine learning algorithms was evaluated for the clinical endpoint LRC. Figure 3 shows the resulting concordance indices (C-Index) for the validation cohort.

The considered learning algorithms achieved in general a high prognostic performance on the validation cohort. The best single performances were obtained by the MSR-RF (C-Index: 0.71, 95% confidence interval [0.62–0.83]), the BT-CIndex (C-Index: 0.71, [0.62–0.82]), the BT-Weibull (C-Index: 0.70, [0.60–0.82]) and the BT-COX (C-Index: 0.70, [0.59–0.81]) algorithms, all in combination with the Spearman feature selection method.

**Figure 4:**
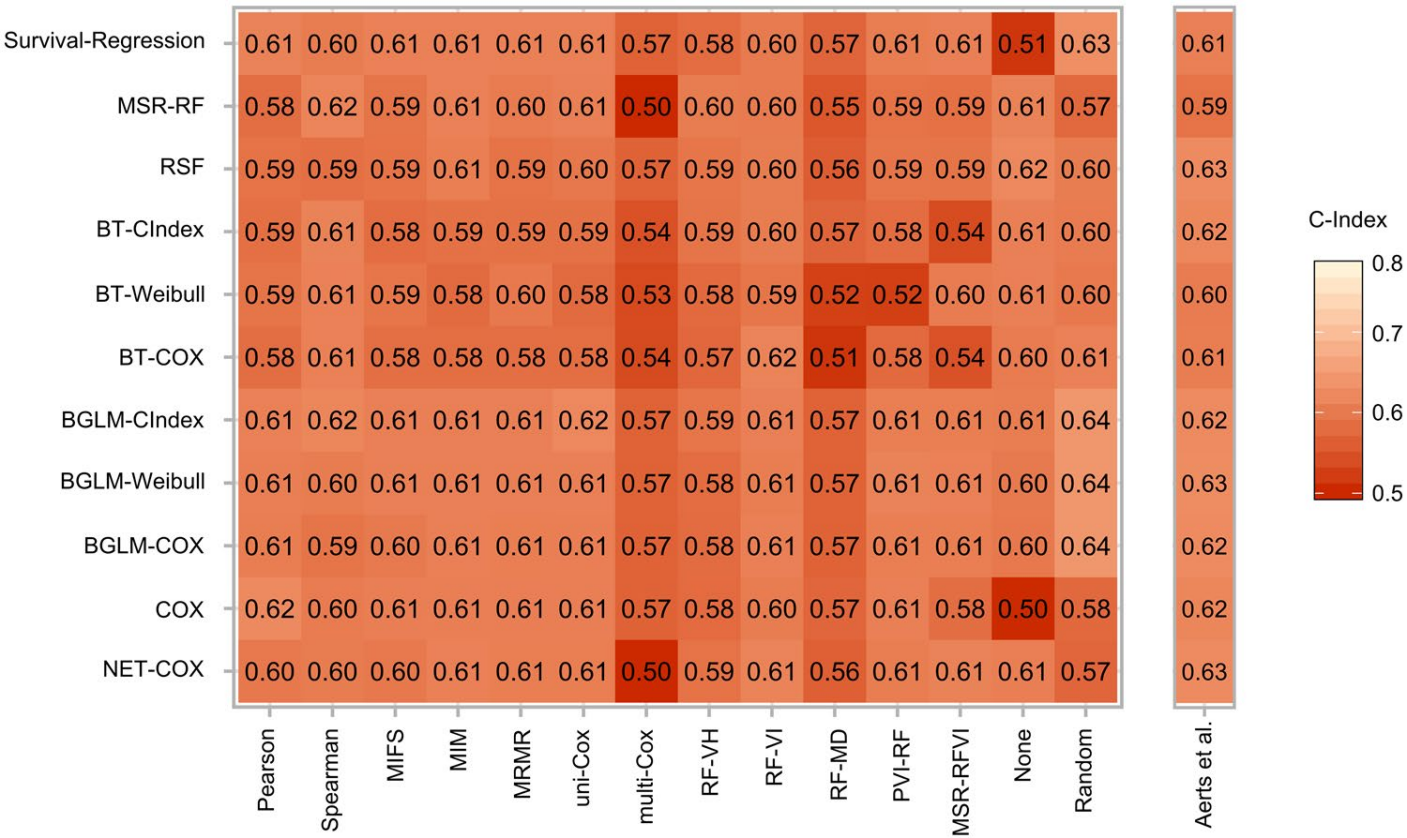
Heatmap depicting the concordance indices depending on the feature selection method (rows) and learning algorithm (columns) for the validation cohort as well as the Aerts *et al*.^5^ signature for overall survival.

For OS, the performance was in general lower in comparison to LRC and similar between the different learning algorithms (Fig. 4). The highest single prediction performances were obtained by the BGLM-CIndex (C-Index: 0.64, [0.53–0.71]), the BGLM-Weibull (C-Index: 0.64, [0.52–0.70]) and the BGLM-COX (C-Index: 0.64, [0.51–0.68]), all in combination with the random feature selection. The resulting C-Index for the exploratory cohort for both clinical endpoints are shown in Supplementary Figures S4 and S5, respectively.

### Median performance of feature selection and machine learning methods

For LRC, the median performance of the learning algorithms over all feature selection methods was in general similar. The highest median performances were obtained by the RSF (C-Index: 0.64 ± 0.03, median ± std), the MSR-RF (C-Index: 0.64 ± 0.04) followed by the NET-COX (C-Index: 0.63 ± 0.04) and the BGLM-CIndex (C-Index: 0.63 ± 0.03). The highest performance of the feature selection methods was achieved by the Spearman correlation coefficient (C-Index: 0.68 ± 0.01). Further methods that performed well were: MRMR (C-Index: 0.65 ± 0.01), MIFS (C-Index: 0.64 ± 0.01), multi-Cox (C-Index: 0.64 ± 0.01), PVI-RF (C-Index: 0.63 ± 0.01) and RF-VI (C-Index: 0.63 ± 0.01).

For OS, the highest median performances were shown by the BGLM-COX (C-Index: 0.61 ± 0.02) and the Survival-Regression (C-Index: 0.61 ± 0.03) algorithm. The performance of feature selection methods was in general similar between the methods. The highest median performances were achieved by the: MIM (C-Index: 0.61 ± 0.01), MRMR (C-Index: 0.61 ± 0.01), None (C-Index: 0.61 ± 0.04) and uni-Cox (C-Index: 0.61 ± 0.01) feature selection methods.

### Robustness of developed radiomic signatures

The features selected into the signature by multi-Cox and uni-Cox feature selection achieved the highest average ICC values for LRC (both ICC: 0.95). The signature determined by spearman feature selection showed a good feature robustness (ICC: 0.69). For OS the highest average ICC values were achieved for Pearson (ICC: 0.96). The lowest average ICC value was shown for the MSR-RFVI feature selection (ICC: 0.86). The average ICC values for both endpoints are depicted in Supplementary Table S6.

### Kaplan-Meier survival analyses

**Figure 5:**
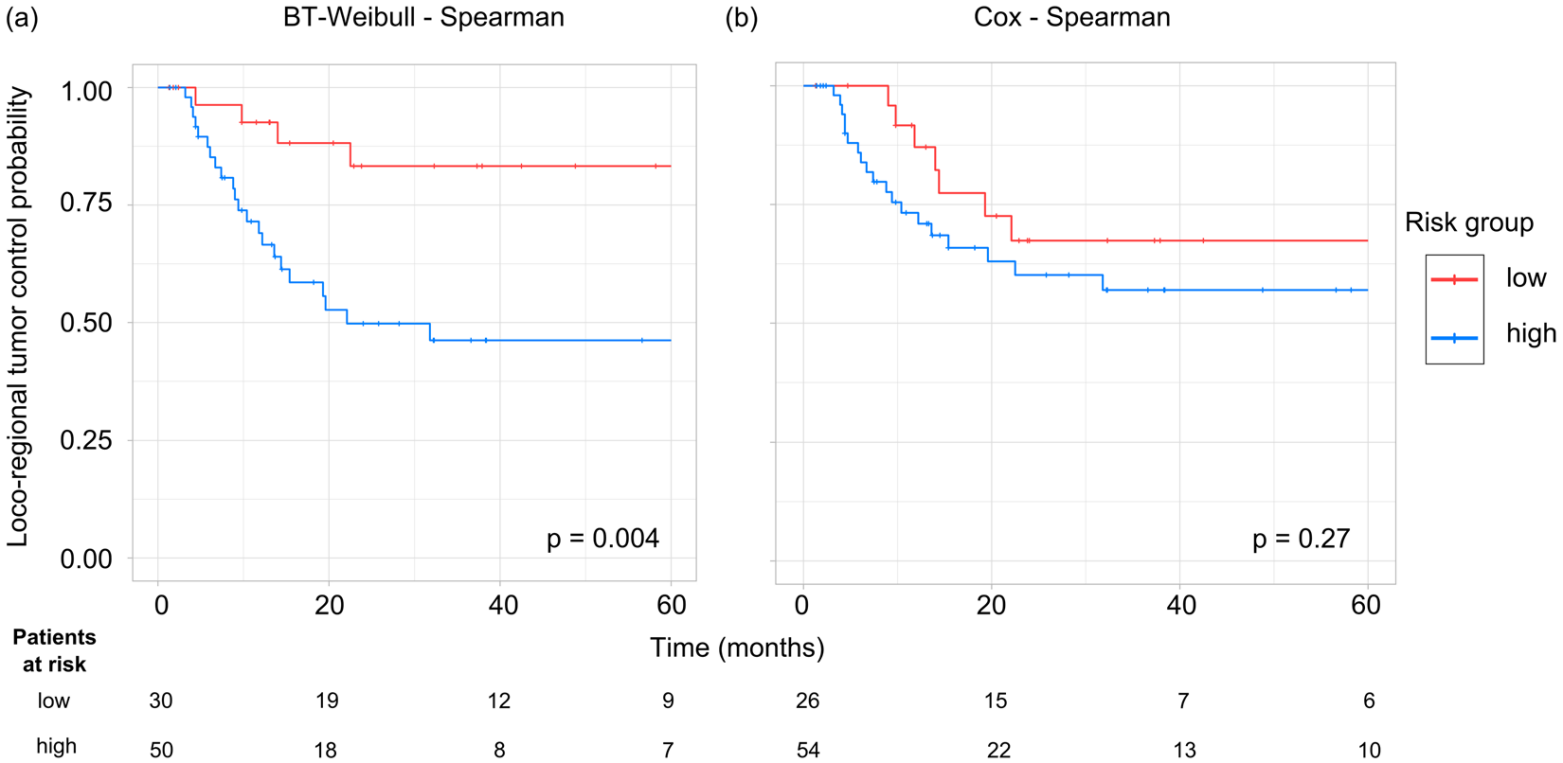
Examples of Kaplan-Meier estimates of loco-regional tumour control for patients of the validation cohort stratified into a low and a high risk group based on a cut-off value determined on the exploratory cohort. (**a**) The BT-Weibull model in combination with Spearman feature selection showed a significant patient stratification as well as a high predictive performance (C-Index: 0.71). (**b**) The Cox model in combination with Spearman feature selection achieved a high predictive performance (C-Index: 0.68) but the difference in LRC between low and high risk group was not significant.

For each combination of feature selection method and learning algorithm, patients were stratified into a low and a high risk group based on the predicted risk of the radiomics signature on the exploratory cohort. The resulting cut-off values were applied to the validation cohort. For LRC the best stratification result on the validation cohort was achieved by the Survival-Regression method trained by the Spearman signature (p = 0.004). The four best performing learning algorithms in combination with Spearman feature selection were able to stratify the patients into a low and high risk group with a significant difference in LRC, confirming the applicability of each model (MSR-RF: p = 0.008, BT-Weibull: p = 0.004 (Fig. 5a), BT-COX: p = 0.023, BT-CIndex: p = 0.032).

However, different models with a high predictive performance were not able to stratify the patients into two groups with significantly different LRC on the validation cohort, *e*.*g*., the Cox model in combination with Spearman feature selection (C-Index: 0.68, p = 0.27, Fig. 5b), whereas models which showed a low predictive performance could stratify patient into two significantly different groups, *e*.*g*., the Cox model trained with the signature determined by the Pearson algorithm (C-Index: 0.58, p = 0.047).

**Figure 6:**
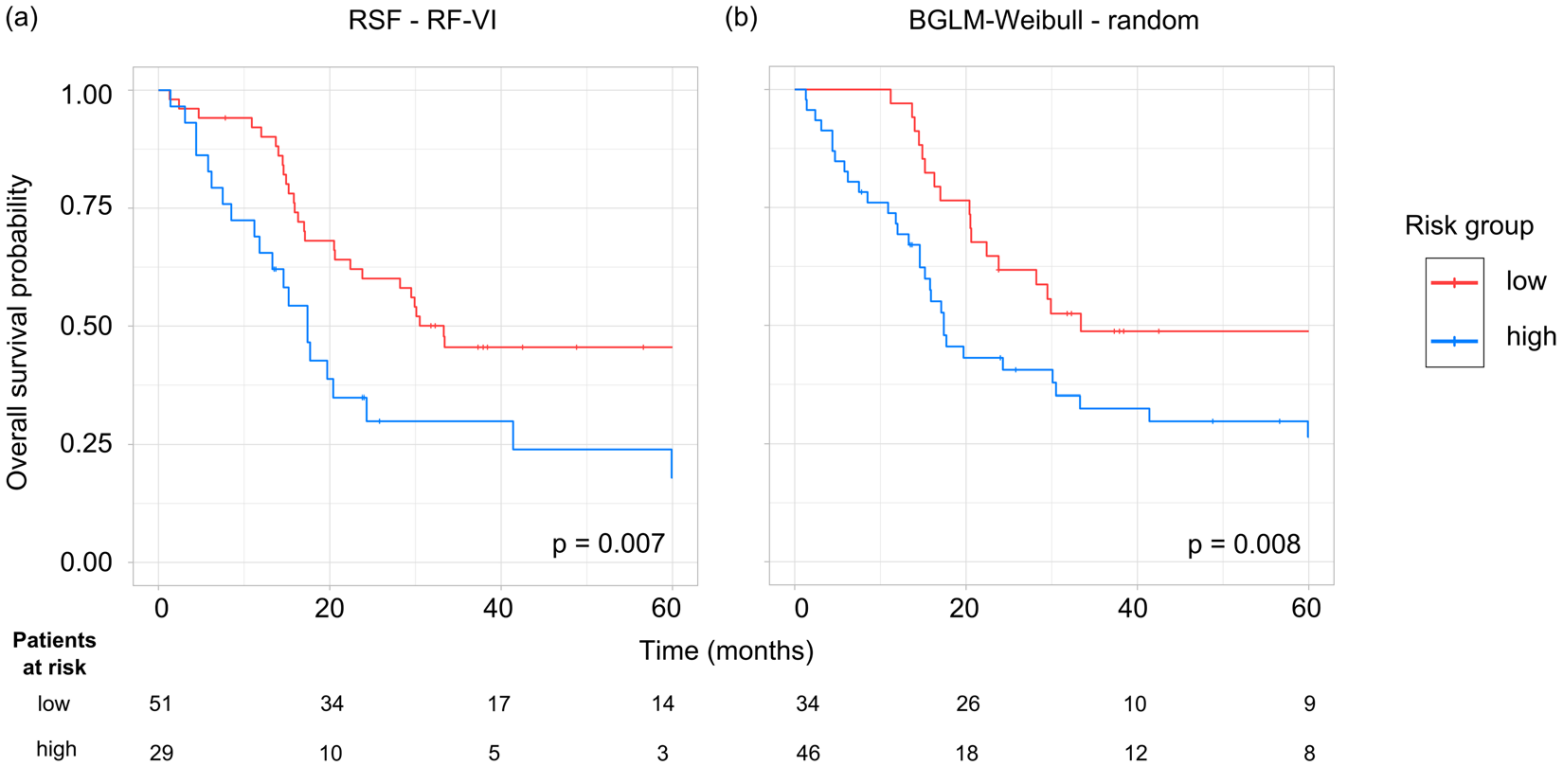
Examples of Kaplan-Meier estimates of overall survival for patients of the validation cohort stratified into a low and a high risk group based on the cut-off determined on the exploratory cohort. (**a**) The RSF model in combination with RF-VI feature selection achieved the most significant patient stratification result although the predicate performance was only moderate (C-Index: 0.60). (**b**) The BGLM-Weibull model in combination with random feature selection achieved a high predictive accuracy (C-Index: 0.64) as well as a significant patient stratification.

For OS, the best stratification result was obtained for the RSF trained with the signature obtained by the RF-VI algorithm (p = 0.007, Fig. 6a). Again, this learning algorithm achieved only a moderate predictive performance (C-Index: 0.60). The BGLM-Weibull in combination with random feature selection achieved a high performance (C-Index: 0.64) as well as a significant patient stratification (p = 0.008, Fig. 6b).

The Kaplan-Meier estimates of the exploratory cohort for the shown examples are depicted in Supplementary Figures S7 and S8 for LRC and OS, respectively. The *p*-values of the log-rank test for all combinations of feature selection methods and learning algorithms on the validation cohort for both clinical endpoints are depicted in Supplementary Figures S10 and S11, respectively.

### Evaluation of Aerts signature

**Figure 7:**
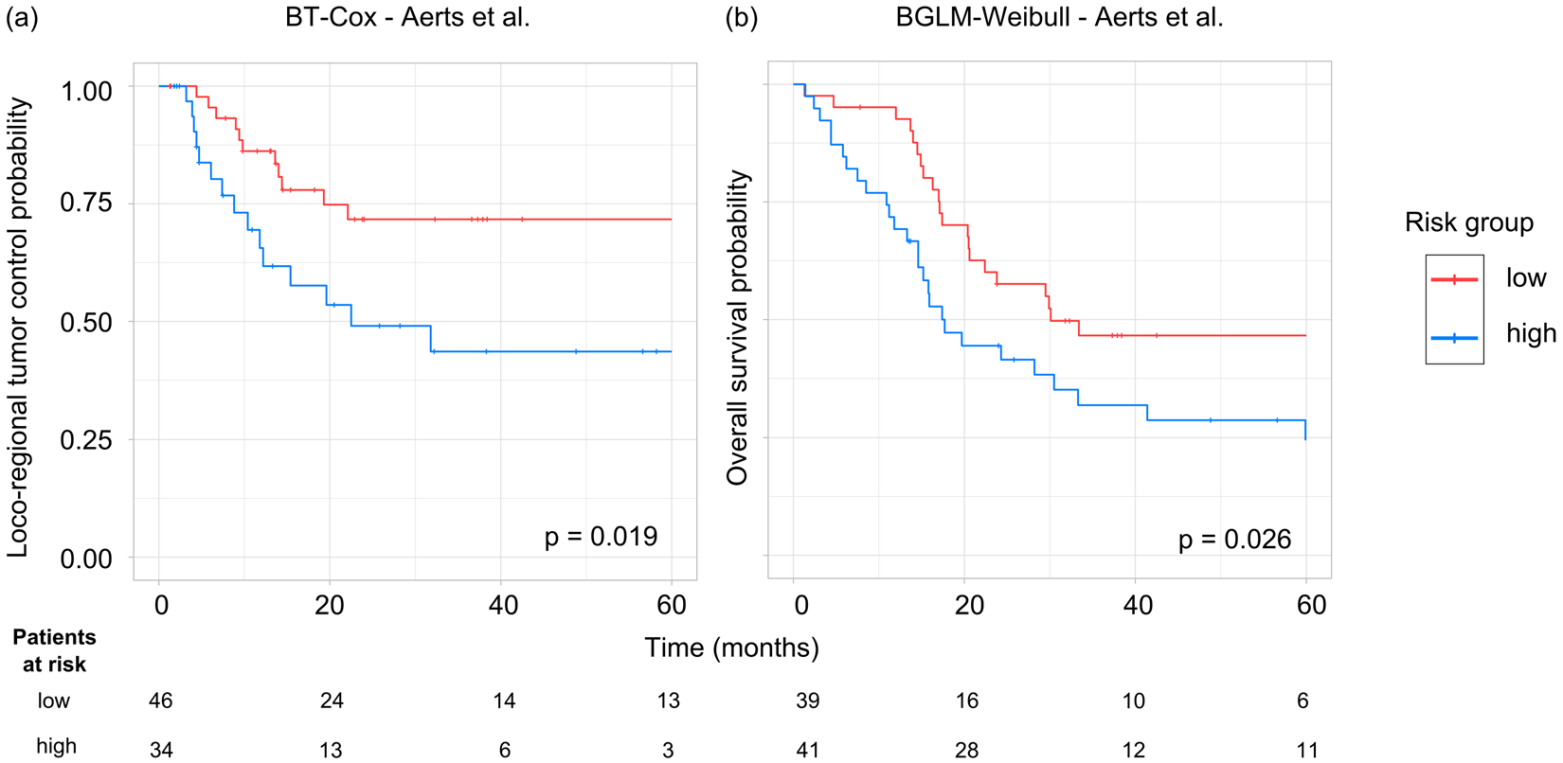
Examples of Kaplan-Meier estimates for (**a**) loco-regional tumour control and (**b**) overall survival for patients of the validation cohort stratified into a low and a high risk group by the cut-off determined on the exploratory cohort. The Aerts *et al*.^5^ signature in combination with the BT-Cox and the BGLM-Weibull model showed a significant patient stratification as well as a high performance (C-Index: 0.65 and C-Index: 0.63, respectively).

The signature by Aerts *et al*.^5^ showed a good performance on the validation cohort for LRC in combination with different learning algorithms. The highest predictive performance could be achieved by the BT-COX (C-Index: 0.65, [0.56–0.76]) and by the BT-Weibull (C-Index: 0.64, [0.55–0.75]) (Fig. 3). From the best performing models (C-Index: 0.71) trained with the signatures determined by the Spearman feature selection, the BT-CIndex model achieved a significantly improved accuracy compared to the BT-COX model trained with the Aerts signature (p < 0.001). For OS the highest performance was achieved by the RSF (C-Index: 0.63, [0.54–0.70]), BGLM-Weibull (C-Index: 0.63, [0.55–0.72]), and NET-Cox (C-Index: 0.63, [0.54–0.71]; Fig. 4). Patients were stratified into a low and a high risk group based on the Aerts *et al*.^5^ signature trained by the BT-Cox model for LRC and the BGLM-Weibull model for OS (Fig. 7). In the validation cohort, both cut-off values could stratify patients with significant differences in LRC (p = 0.019) and OS (p = 0.026), respectively. The Kaplan-Meier estimates of the exploratory cohort for the shown examples are depicted in Supplementary Figure S9.

## Discussion

The identification of suitable feature selection methods and learning algorithms is a critical step to develop accurate radiomics models. We therefore performed a systematic evaluation of different feature selection methods and learning algorithms in predicting LRC and OS for patients with locally advanced HNSCC. All algorithms were able to deal with continuous time-to-event survival data and have not been investigated previously.

In general, the systematic evaluation showed a good predictive performance for LRC. However, there was no method which noticeably outperformed all others. Instead, a subset of different feature selection methods and learning algorithms led to similar results. This indicates that a wide range of different methods are useful and should be considered for further radiomics analyses. Moreover, applying multiple methods may decrease the influence of random effects which may occur in selecting one single approach. Furthermore, the evaluation showed that the performance differences between the learning algorithms were smaller than between the feature selection methods. In line with Parmar *et al*.^10,11^, this indicates that feature selection is more important in the process of developing an accurate radiomics model. In contrast to LRC, the predictive performance for OS was generally lower. This may occur since the cause of death does not necessarily have to be related to cancer, and due to the corresponding increase in “noise” on the outcome. Furthermore the best performances were achieved by the BGLM-CIndex, -Weibull and -Cox models in combination with the random feature selection. One explanation for the good performance of random feature selection may be that the hyper-parameter optimisation combined with feature selection, which is performed internally by several of the classifiers, leads to a better model accuracy. However, the results on the training set (Supplementary Figure S5) show that the train error is actually greater than the test error for the cases with high prognostic performance. This indicates that the resulting validation performance may be a statistical fluke. A significant difference in LRC was found between patients stratified into a low and a high risk group using the best performing models, which confirms their clinical potential. However, the results strongly depend on the process of selecting the stratification cut-off, and not necessarily on the performance of the risk model. This was the case for multiple models which predicted risk well, yet did not lead to a stratification of patients to risk groups with significant differences in loco-regional tumour recurrence. Still, the applied cut-off selection process, which is based on different bootstrap samples, may lead to more robust results compared to, *e*.*g*., a median cut-off. For instance, the cut-off determination based on bootstrap samples led to more significant results (n = 30) than the median cut-off (n = 20) on the validation cohort for LRC.

The signature obtained by the Spearman feature selection method achieved a higher validation performance than the signature by Aerts *et al*.^5^, which, however, was developed on a dataset of lung cancer patients. The features included in the signature by Aerts *et al*.^5^ were selected based on their stability across test-retest image scans, multiple tumour delineations and 100 bootstrap samples on the exploratory cohort. They represent the single best feature from each of four feature groups (statistical, shape, texture and wavelet features). In contrast, our radiomics signature for the Spearman method consisted mainly of features extracted from transformed images (*e*.*g*., wavelet), leading to more sensitivity to image perturbations, *e*.*g*., image rotations and translations. However, models trained using Spearman method showed the highest validation performance which indicates that those image perturbations had in the end a limited effect on the model accuracy.

The robustness of the signatures obtained by the different feature selection methods was in general very high against image rotations and translations. We included 293 HNSCC patients from different institutions in our study, resulting in a highly heterogeneous dataset, which captures the variability between different CT settings and reconstruction parameters usually affecting the model accuracy^35,36^. Therefore the obtained signatures might be less biased from single-centre selection effects and thereby more generalisable and robust. Furthermore the stability of feature selection methods was not assessed directly, as we observed that selected features in a particular bootstrap varied greatly from one bootstrap to the next, *i*.*e*., we found low overlap. Therefore we aggregate feature ranks and select features based on rank and occurrence using an adaptation of the enhanced Borda score^29^ which may also increase the stability. To further enhance its robustness, feature stability information, *e*.*g*., from test re-test datasets, could be included in the future. To improve the comparability and applicability of radiomics signatures, image processing should be done according to the recommendations of the imaging biomarker standardization initiative^23^.

The prognostic performance of radiomic analyses may in principle be further improved using the deep-learning approach. In particular convolutional neural networks (CNNs) are able to learn feature representations directly from the imaging data instead of using hand-crafted features. The application of CNNs have already showed promising results in the medical imaging domain^37^ such as image segmentation^38^ or lesion detection^39^. However, only few studies so far investigated the potential of the deep learning approach for radiomics^40^. Paul *et al*.^41^ showed that deep learning features alone did not improve the model accuracy in comparison to traditional quantitative imaging features. Therefore, in the present study we decided to focus on traditional quantitative imaging features because investigation of CNNs still requires substantial fundamental research concerning the application of deep learning in radiomics: Deep learning is usually based on 2D images, which reduces the available information of the tumour. In general 3D-CNNs are possible, however, for such an approach no pre-trained networks are available which requires sufficient more data samples for training to avoid *e*.*g*., model overfitting. A further, unresolved, limitation is the missing ability to handle (censored) continuous time-to-event survival data, which was the main focus of our study. Still, deep-learning is a promising approach for radiomics and requires further investigation. Therefore we are focusing on both extending our curated medical image data set and addressing several open methodological questions to adapt deep learning to radiomic risk modelling in the future.

We developed a radiomics modelling framework (RMF) to perform unbiased automatic radiomics analyses. Before feature selection, unsupervised feature clustering was performed to reduce and group redundant features. The hierarchical clustering process depends on the cut-off threshold. The cut-off height was set manually, based on considerations for the resulting number of clusters and likely redundancy. The selection of a different cut-off height may result in slightly different findings. Furthermore, it is conceivable to perform a model-based feature pre-selection on the exploratory cohort. For instance, a univariate Cox regression model may be used to remove non-informative features, followed by feature clustering to optimise the cut-off value at the beginning of the processing chain.

After the feature selection process, automatic hyper-parameter optimisation was performed by the RMF, which has not been considered in most of the previously radiomics studies. One reason is the computational resources required to optimise the model parameters. To limit these resources, we defined hyper-parameter ranges for each algorithm with the aim to reduce the parameter space. The parameter space was defined based on prior knowledge, *e*.*g*., the maximum signature size was derived by the number of events, *i*.*e*., 10 events per predictor variable as well as identifying those settings that led to balanced performance in cross-validation of the exploratory cohort. A further time reduction could be achieved by replacing the exhaustive grid search optimisation by a random search strategy^42^. Nevertheless, the automatic hyper-parameter optimisation at the beginning of the model training adjusts the model to the specific prediction task, which may improve the prediction accuracy and reduce the influence of model overfitting. This is particularly important for the more complex models (*e*.*g*., BT-Weibull, BT-Cox and MSR-RF), as the choice of their hyper-parameters influences how well they can learn the underlying data distribution.

Based on our systematic evaluation of machine-learning algorithms for continuous time-to event survival data we conclude that the Cox model can be used as a baseline predictive model. The Cox model, despite its simplicity was able to achieve results comparable to the more complex models. In addition, the tree based methods (BT-Cox, RSF, MSR-RF) or full-parametric models like BT-Weibull and the boosted gradient linear model (BGLM-Cox) should be considered. In the case of feature selection methods we recommend the Spearman correlation coefficient and mutual information based methods (MRMR, MIM, MIFS). Multivariate-Cox feature selection as well as the random forest based method (RF-VI, PVI-RF) led to acceptable performance and may also be evaluated.

In conclusion, a wide range of available machine-learning methods appears useful in future radiomics studies. The application of suitable feature selection methods and learning algorithms is an important step to increase the robustness of future radiomics studies. Furthermore, it helps to standardise the methods within the radiomics processing chain towards more stable and relevant clinical risk models.

### Data availability

The datasets used and analysed during the current study are available from the corresponding author on reasonable request.

## Electronic supplementary material

Supplementary information
