# Supplementary material for: A comparative study of machine learning methods for time-to-event survival data for radiomics risk modelling
Source: Sci Rep. 2017 Oct 16;7:13206. doi: 10.1038/s41598-017-13448-3 (PMC5643429; doi:10.1038/s41598-017-13448-3)
Supplement: Supplementary file 1 — Supplementary information [file 41598_2017_13448_MOESM1_ESM.pdf]

A comparative study of machine learning methods for time-to-event survival data for radiomics risk modelling

Stefan Leger[1,2], Alex Zwanenburg[1,2,8,9], Karoline Pilz[1,2,8,10], Fabian Lohaus[1,2,8,10], Annett Linge[1,2,8,10], Klaus Zöphel[12,13], Jörg Kotzerke[12,13], Andreas Schreiber[14] Inge Tinhofer[3,15], Volker Budach[3,15], Ali Sak[4,16], Martin Stuschke[4,16], Panagiotis Balermpas[5,17], Claus Rödel[5,17], Ute Ganswindt[18-20] Claus Belka[6,18-20], Steffi Pigorsch[6,21], Stephanie E. Combs[6,21,22], David Mönnich[7,23], Daniel Zips[7,23], Mechthild Krause[1,2,8,10,11], Michael Baumann[1,2,8-11], Esther G.C. Troost[1,2,8,10,11], Steffen Löck[1,2,10,*] and Christian Richter[1,2,10,11,*]

[1] OncoRay - National Center for Radiation Research in Oncology, Faculty of Medicine and University Hospital Carl Gustav Carus, Technische Universität Dresden, Helmholtz-Zentrum Dresden – Rossendorf, Dresden, Germany

German Cancer Research Center (DKFZ), Heidelberg and German Cancer Consortium (DKTK) partner sites:

[2] Dresden, [3] Berlin, [4] Essen, [5] Frankfurt, [6] Munich, [7] Tübingen, Germany

[8] National Center for Tumor Diseases (NCT), partner site Dresden, Germany

[9] German Cancer Research Center (DKFZ), Heidelberg, Germany

[10] Department of Radiotherapy and Radiation Oncology, Faculty of Medicine and University Hospital Carl Gustav Carus, Technische Universität Dresden, Dresden, Germany

[11] Helmholtz-Zentrum Dresden – Rossendorf, Institute of Radiooncology - OncoRay, Dresden, Germany

[12] Department of Nuclear Medicine, Faculty of Medicine and University Hospital Carl Gustav Carus, Technische Universität Dresden, Dresden, Germany

[13] Helmholtz-Zentrum Dresden-Rossendorf, PET Center, Institute of Radiopharmaceutical Cancer Research, Dresden, Germany

[14] Clinic of Radiation Oncology, Teaching Hospital Dresden - Friedrichstadt, Technische Universität Dresden, Dresden, Germany

[15] Department of Radiooncology and Radiotherapy, Charité University Hospital, Berlin, Germany

[16] Department of Radiotherapy, Medical Faculty, University of Duisburg-Essen, Essen, Germany

[17] Department of Radiotherapy and Oncology, Goethe-University Frankfurt, Germany;

[18] Heidelberg Ion Therapy Center (HIT), Department of Radiation Oncology, University of Heidelberg Medical School, Germany

[19] Department of Radiation Oncology, Ludwig-Maximilians-Universität, Munich, Germany;

[20] Clinical Cooperation Group, Personalized Radiotherapy in Head and Neck Cancer, Helmholtz Zentrum, Munich, Germany

[21] Department of Radiation Oncology, Technische Universität München, Germany;

[22] Institute of Innovative Radiotherapy (*i*RT), Helmholtz Zentrum München, Oberschleißheim, Germany

[23] Department of Radiation Oncology, Faculty of Medicine and University Hospital Tübingen, Eberhard Karls Universität Tübingen, Germany

* These authors share senior authorship.

Corresponding author: Stefan Leger (email: Stefan.Leger@oncoray.de)

Supplementary Table S1: Patient characteristics of exploratory and validation cohorts.

Variable	Exploratory cohort	Validation cohort	p-value
Gender (male/female)	181 / 32	70 / 10	0.58*
Age (median, range, in years)	59.0 (39.0 – 81.9)	54.0 (37.0 – 74.0)	0.005**
TNM Staging<br>T Stage 1 / 2 / 3 / 4 / missing	3 / 24 / 53 / 133 / 0	3 / 9 / 27 / 40 / 1	0.19*
N stage 0 / 1 / 2 / 3 / missing	25 / 8 / 166 / 14 / 0	10 / 8 / 57 / 4 / 1	0.19*
UICC stage 2010<br>I / II / III / IV / missing	0 / 0 / 13 / 139 / 61	1 / 2 / 9 / 68 / 0	0.096*
Tumor volume (median, range, in cm^3)	27.64 (0.27 – 276.31)	34.85 (2.71 – 244.79)	0.19**
Dose (median, range, in Gy)	72.0 (67.8 – 76.8)	72.0 (69.0 – 76.8)	< 0.001**
HPV16 DNA<br>negative / positive / missing	164 / 27 / 22	39 / 5 / 36	0.63*

***** X^2 test, ** Wilcoxon-Mann-Whitney test

Supplementary Methods S2: Feature selection methods

The mathematical notation for the selection methods and learning algorithms is defined as follows: An observed random variable is defined as $Z = (X, Y)$ where $X = (X_1, \dots, X_p)$ defines the *p*-dimensional covariate (feature) vector and *Y* denotes the time-to-event survival outcome. The survival outcome $Y = (t, \delta)$ consists of a survival time t at which an event occurred ($\delta = 1$) or the observation was censored ($\delta = 0$).

- Pearson correlation

The Pearson correlation coefficient is a measure of linear dependency between two random variables. The correlation coefficient *r* for a feature $x \in X$ and the corresponding outcome *Y* is defined as [1]

$$r = \frac{\sum_{i=1}^{n}(x_i - \bar{x})(y_i - \bar{Y})}{\sqrt{\sum_{i=1}^{n}(x_i - \bar{x})^2 \sum_{i=1}^{n}(y_i - \bar{Y})^2}}. \quad (1)$$

In the case of time-to-event survival data, only those times y_i for which an event occurred $(\delta_i = 1)$ are taken into account. The correlation coefficient *r* ranges from -1 and +1, where 1 signifies perfect linear correlation, 0 no correlation, and −1 perfect anti-correlation. For the implementation in the radiomics modelling framework (RMF), the R package “stats” version 3.3.1 was used.

- Spearman correlation

The Spearman correlation coefficient provides a non-parametric measure of correlation between two variables [2]. As with Pearson correlation, for time-to-event survival data the Spearman correlation is calculated using only those observations where an event occurred. For the implementation in the RMF, the R package “stats” version 3.3.1 was used.

- Mutual information maximization

The mutual information maximization (MIM) method estimates the relevance of feature $x \in X$ for the corresponding outcome Y using a linear approximation based on the correlation ρ such that the mutual information *I* is estimated as

$$I(x, Y) = -\frac{1}{2} ln(1 - \rho(x, Y)^2). \quad (2)$$

In the case of survival outcome data $\rho(x, Y) = 2 \cdot (\text{C-Index} - 0.5)$ is based on the concordance index (C-Index) [3,4], including a correction for C-Index<0.5. For mutual information of continuous data, Spearman correlation is used.

- Mutual information feature selection

The mutual information feature selection (MIFS) algorithm [5] is based on a greedy search and selects a subset of features $S \in X$ which maximises the objective function Ω:

$$\Omega = \underset{x \in X}{\operatorname{argmax}} \; I(x, Y) \; + \; \beta \sum_{s_j \in S} I(x, s_j). \tag{3}$$

Here $I(x_a, x_b)$ is the mutual correlation between features x_a and x_b, as before. We use the setting $\beta = 1$ after recommendations by Battiti et al.

- Minimum redundancy maximum relevance

The minimum redundancy maximum relevance (MRMR) algorithm [6] combines two constraints: maximal mutual information between the features in feature subset $S \in X$ and the outcome *Y*, combined with minimal redundancy between the features in *S*. This is done by selecting the feature that maximizes Ω by an incremental search method, which is based on the mutual information *I*,

$$\Omega = \underset{x \in X \setminus S}{\operatorname{argmax}} \, I(x, Y) - \frac{1}{|S|} \sum_{s_j \in S} I\left(x, s_j\right). \tag{4}$$

- Univariate- and multivariate-Cox-regression

The Cox proportional hazard regression model is trained for each feature (univariate) or a subset of features (multivariate) to predict outcome using a 2-fold cross validation scheme which was repeated 20 times on the exploratory cohort. The resulting features are ranked according to the concordance index of the predictive performance of the univariate or multivariate model. For the implementation in RMF, the R package “survival” [7] was used.

- Random forest minimal depth

The random forest minimal depth (RFMD) [8,9] is a variable importance algorithm that assesses feature importance by looking at the distance (depth) of each feature relative to the root node over all trees. The algorithm assumes that features, which occur at low depths, are

more important for the model than those in distant nodes. The R implementation of the package “randomForestSRC” in version 2.4.1 was used [10].

- Random forest variable hunting

The random forest variable hunting RF-VH [10] algorithm uses training data from a stratified *k*-fold subsampling to fit a forest by *m* randomly selected features. The *m* features are ordered by increasing minimal depth and are added sequentially until the joint variable importance (VIMP) no longer increases. The VIMP is calculated by permuting a feature (i.e. noising it up) and then calculating the change in prediction error, between the original forest and the noised-up forest predictor. The process is repeated *n* times and features are ranked by average minimal depth. The R implementation of the package “randomForestSRC” in version 2.4.1 was used [10].

- Random forest variable importance

The random forest variable importance (RF-VI) [10] algorithm is similar to the RF-VH. However, features are ranked by the VIMP score, described above. The R implementation of the package “randomForestSRC” in version 2.4.1 was used [10].

- Maximally selected rank statistics random forest variable importance

The maximally selected rank statistics random forest variable importance (MSR-RFVI) [11] algorithm computes for each candidate covariate (i.e. feature) the maximally selected rank statistics, as follows. A split point is considered optimal if the separation of the survival curves in two groups is maximised. The linear rank statistic for a split point μ is the sum of all log-rank scores $a_1, \dots, a_n$ in the group with $x_i \leq \mu$, $x \in X$,

$$S_{n\mu} = \sum_{i=1}^{n} 1_{\{x_i \leq \mu\}} \, a_i, \tag{5}$$

$$a_i = \delta_i - \sum_{j=1}^{\gamma_i(T)} \frac{\delta_j}{(n - \gamma_j(T) + 1)}. \quad (6)$$

Here $T = (t_1, \ldots, t_n)$ are the survival times, δ is the censoring indicator and $\gamma_j(T) = \sum_{i=1}^{n} 1_{\{T_i \leq T_j\}}$ is the number of observations with survival time up to T_j. To compare different splits, the score test statistic is used,

$$T_{n\mu} = \frac{S_{n\mu} - E_{H_0}(S_{n\mu}|a, X)}{\sqrt{Var_{H_0}(S_{n\mu}|a, X)}}, \quad (7)$$

where $E_{H_0}$and Var_{H_0} define the expectation and variance under the null hypothesis. The null hypothesis of no influence of a split by the cut point μ on the distribution of Y is $H_0 \colon P(Y \leq y|X \leq \mu) = P(Y \leq y|X > \mu)$ for all μ and all y. The obtained *p*-value for the maximally selected rank statistic is used to rank each covariate. For the implementation, the R package “ranger”, version 0.6.0 [12] was used.

- Permutation variable importance random forest

The permutation variable importance random forest (PVI-RF) [13] algorithm partitions the data randomly into two sets of equal size. Each set is used to construct a random forest. The two forests are used to compute variable importance of the hold-out observations for each covariate. The null distribution $\widehat{F}$ is constructed afterwards based on variables that are likely non-relevant (*i.e.*, with negative or zero importance scores). Based on $\widehat{F}$, a *p*-value for a covariate $x \in X$ is derived as,

$$p_x = 1 - \widehat{F}(x). \quad (8)$$

Finally the VIMP are ranked according their corresponding *p*-value. For the implementation, the R package “ranger”, version 0.6.0 [12] was used.

Supplementary Methods S3: Learning algorithms

- Cox proportional hazards model

The hazard function $\lambda(t|X)$ of the Cox proportional hazards model (Cox) is defined as [14]

$$\lambda(t|X) = \lambda_0(t)e^{X\beta}, \tag{9}$$

where $\lambda_0(t)$ is the baseline hazard function and β the p-dimensional vector of regression coefficients. The regression coefficients β are estimated by maximising the partial log-likelihood:

$$LL(\beta) = \sum_{i=1}^{n} \delta_i \left(X\beta - \log\left(\sum_{k:t_k \geq t_i} \exp(X_k\beta) \right) \right). \tag{10}$$

For the implementation in the RMF, the R package “survival” [7] with version 2.4-10 was used as well as the following parameters for the hyper-parameter optimization:

Parameter	Values
signature size	2,3,4,5,7,10

- Regularized Cox proportional hazard model

The regularized Cox proportional hazard model (NET-Cox) is based on a Cox model and a convex combinations of l_1 and l_2 penalties [15]. The penalised constraint $P_\alpha(\beta)$ is a mixture of the l_1 (lasso) and l_2 (ridge regression) penalty, which is used to maximise the scaled log partial likelihood

$$LL(\beta) = \underset{\beta}{\operatorname{argmax}} \left[\frac{2}{n} \left(\sum_{i=1}^{n} \delta_i \left(X\beta - \log\left(\sum_{k:t_k \geq t_i} \exp(X_k\beta) \right) \right) \right) - \lambda P_\alpha(\beta) \right], \tag{11}$$

$$\lambda P_\alpha(\beta) = \lambda \left(\alpha \sum_{j=1}^{p} |\beta_j| + \frac{1}{2}(1-\alpha) \sum_{j=1}^{p} \beta_j^2 \right). \tag{12}$$

The R package “glmnet” version 2.0-5 was used for the implementation [16]. For hyper-parameter optimization the following parameters were used:

Parameter	Values
signature size	2,3,4,5,7,10
α	0.0-1.0, step size 0.2
λ	lambda.min*,lambda.1se**

*minimum mean cross-validated error, ** Error within one standard error of the minimum

- Boosting gradient linear and boosted tree models

The aim of the boosting gradient linear models (BGLM) is to learn a functional mapping to find $y = f^*(Y|X,\lambda)$ from data X, Y and a set of model parameters λ. The functional mapping is learned by minimising the loss function Φ of the empirical risk:

$$f^*(Y|X,\lambda) = \underset{f}{\operatorname{argmin}} \sum_{i=1}^{n} \Phi\big(Y, f(X,\lambda)\big), \tag{13}$$

where f is called a base-learner. The gradient boosting algorithm estimates at each iteration $m = 1, \dots, m_{stop}$ the negative gradient $-\frac{\partial \Phi}{\partial f}$ of the loss function and evaluates it at $f^{[m-1]}(X,\lambda), i = 1, \dots, n,$ yielding the negative gradient vector $u^{[m]} := \left(-\frac{\partial \Phi}{\partial f}\left(Y, f^{[m-1]}(X,\lambda)\right)\right)_{i=1,\dots,n}$ for each base-learner. Typically one base-learner for each covariate is used, resulting in *P* vectors of prediction values. Afterwards, the best base-learner is selected and $\dot{u}^{[m]}$ is set equal to the fitted values from the corresponding best learner. Finally the function mapping $f^{[m]} = f^{[m-1]} + \alpha\dot{u}^{[m]}$ is updated, where $0 < \alpha \leq 1$ is a step-length factor. The loss function Φ to be optimised can be specified by Cox's partial likelihood (Cox), the concordance index (CIndex) as well as the Weibull distribution (Weibull). In the case of boosting tree models (BT), regression trees are used as base-learners.

The R package "mboost" [17] version 2.7-0 was used in the RMF. For hyper-parameter optimisation the following parameter were set:

Parameter	Values
signature size	2,3,4,5,7,10
α	0.001,0.01,0.05
λ	lambda.min,lambda.1se
mStop	200

- Random survival forest

The random survival forest (RSF) [18] is an extension of Breiman's random forest algorithm [19] for survival data. The basic idea is to draw *ntree* bootstrap samples from the exploratory cohort. For each sample, a survival tree is trained. For each node of the tree, *mtry* features are randomly selected as splitting candidates. For each splitting candidate, a maximum of *nSplit* split points are randomly chosen among the possible split points. The possible splitting rules for survival data are the *logrank* and the *logrankscore* splitting criteria. Selecting splitting candidates and split points is repeated until either the terminal nodes contains no less than *nodeSize* unique events or the *maxDepth* of the tree is reached. Based on the resulting tree ensemble, cumulative hazard estimates are calculated by combining all information from the *ntree* trees. The R package "randomForestSRC" in version 2.4.1 was used [10]. For hyper-parameter optimisation the following parameters were used:

Parameter	Values
signature size	2,3,4,5,7,10
ntree	2000
mtry	100
nodeSize	25-50
maxDepth	10,15
nSplit	1,2,100
splitRule	logrank,logrankscore

- Maximally selected rank statistics random forest

The maximally selected rank statistics random forest (MSR-RF) [11] algorithm is based on an improved split point criterion to reduce split point selection bias. In MSR-RF a split point is considered optimal if the separation of survival curves in the two groups is maximised. The standard split criterion for the RSF is the *logrank* or the *logrankscore* test statistic. In contrast, the MSR-RF uses either the maximally selected rank statistics (*maxstat*) or the Harrell's C statistics (C) for split point selection. As described above, the covariate with the lowest *p*-value is selected as splitting candidate. If the adjusted *p*-value is not smaller than the threshold α, no splitting is performed. For the implementation, the R package "ranger",

version 0.6.0 [12] and the following parameters for the hyper-parameter optimisation were used:

Parameter	Values
signature size	2,3,4,5,7,10
ntree	2000
mtry	100
nodeSize	25-50, step size 1
minprop	0.1
α	0.1,0.5
splitRule	C, maxstat

- Survival regression model

The survival regression model (SurvivalReg) is a full-parametric model, which provides different survival functions, *e.g.*, Weibull, Exponential, Gaussian. For instance, the Weibull probability density function is given by

$$\mathrm{f}(t) = \frac{\lambda t^{\lambda-1}}{\alpha^{\lambda}} \cdot e^{-\left(\frac{t}{\alpha}\right)^{\lambda}} = h(t) \cdot S(t), \quad (14)$$

which is a combination of the hazard function $h(t)$ and the corresponding survival function $S(t)$. The free parameters λ and α are called shape and scale of the Weibull distribution which were estimated during training. The hazard function is defined as [20],

$$h(t, X, \beta, \lambda) = \lambda e^{-\lambda X \beta}\, t^{\lambda-1}, \quad (15)$$

where the scale parameter $\lambda = 1/\sigma$ is defined. In contrast to the Cox model, for which the baseline hazard is unknown, this full-parametric regression allows for predicting the time-dependent survival probability of each patient. For implementation, the R package “survival”, version 2.4-10 [7] and the following parameters for the hyper-parameter optimization were used:

Parameter	Values
signature size	2,3,4,5,7,10
distribution	weibull, gaussian, exponential

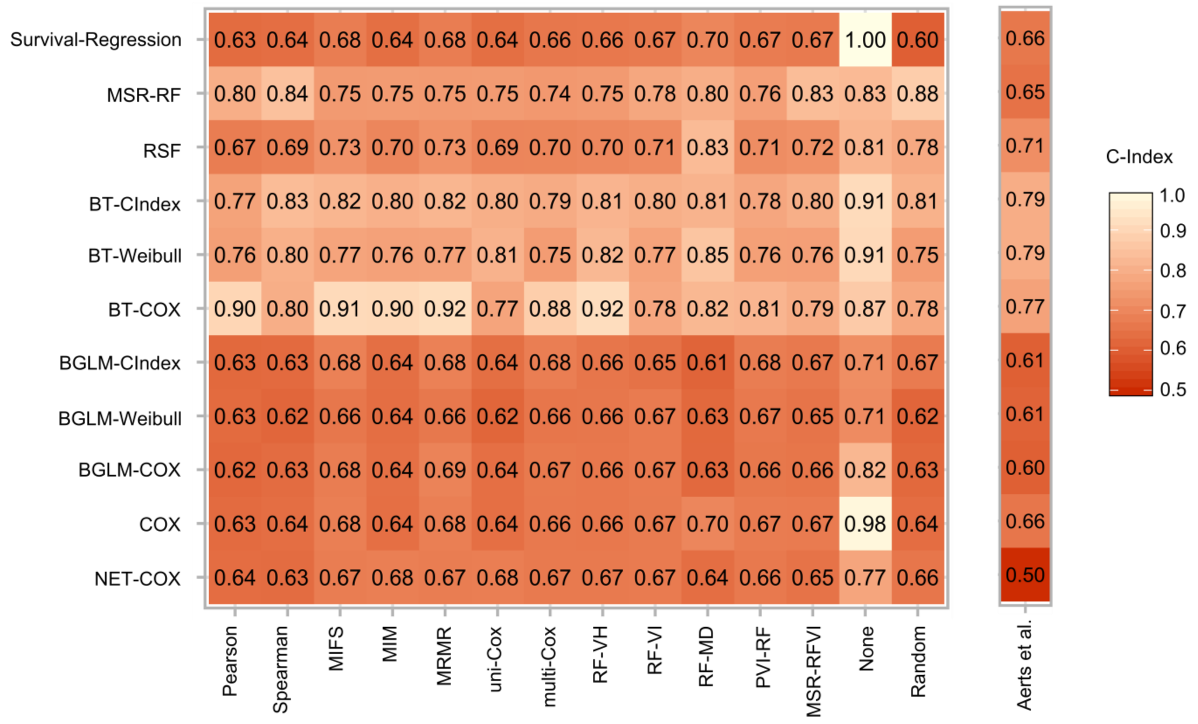

Supplementary Figure S4: Heatmap depicting the concordance indices for every combination of feature selection method (rows) and learning algorithm (columns) for the exploratory cohort as well as the signature by Aerts *et al.* [21] for loco-regional control.

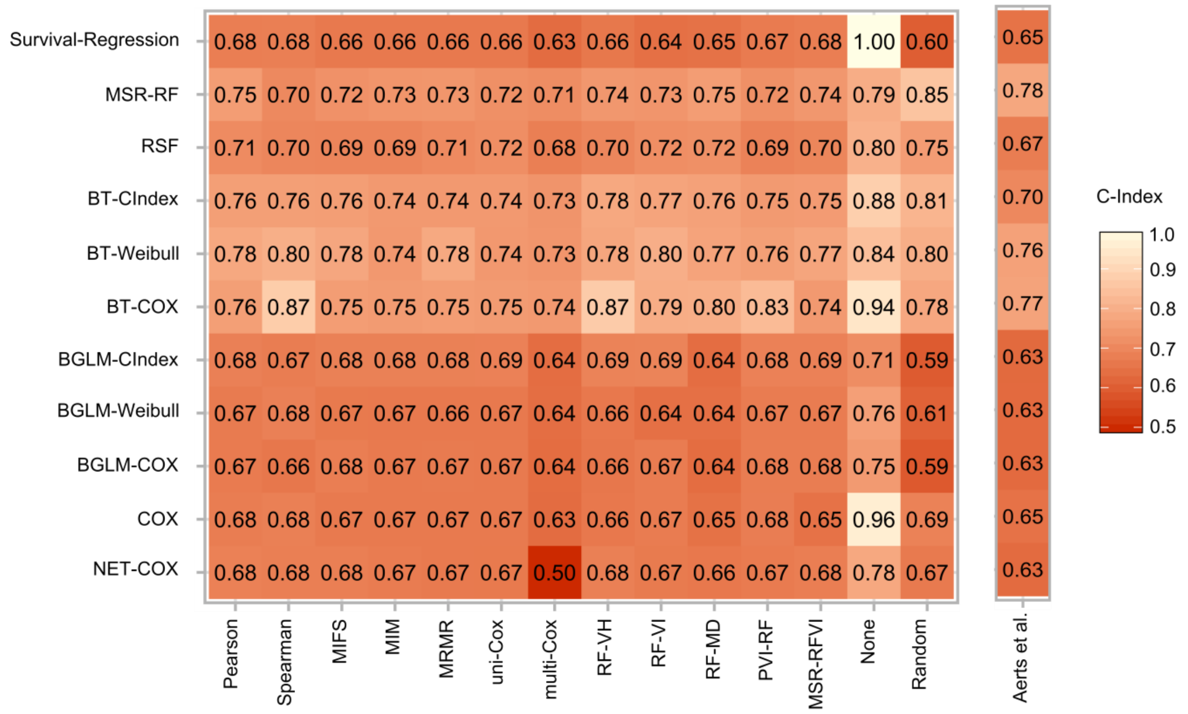

Supplementary Figure S5: Heatmap depicting the concordance indices for every combination of feature selection method (rows) and learning algorithm (columns) for the exploratory cohort as well as the signature by Aerts *et al.* [21] for overall survival.

Supplementary Table S6: Average intra-class correlation coefficient for different image rotations and translations to measure feature robustness of the developed signatures for loco-regional tumour control and overall survival.

Feature selection method	LRC	OS
Pearson	0.88±0.019	0.96±0.006
Spearman	0.69±0.043	0.94±0.007
MIFS	0.76±0.025	0.93±0.013
MIM	0.93±0.024	0.94±0.000
MRMR	0.78±0.032	0.94±0.012
uni-Cox	0.95±0.002	0.94±0.003
multi-Cox	0.95±0.000	0.94±0.000
RF-VH	0.89±0.006	0.94±0.000
RF-VI	0.90±0.006	0.93±0.023
RF-MD	0.87±0.025	0.93±0.010
PVI-RF	0.90±0.053	0.92±0.019
MSR-RFVI	0.89±0.035	0.86±0.000

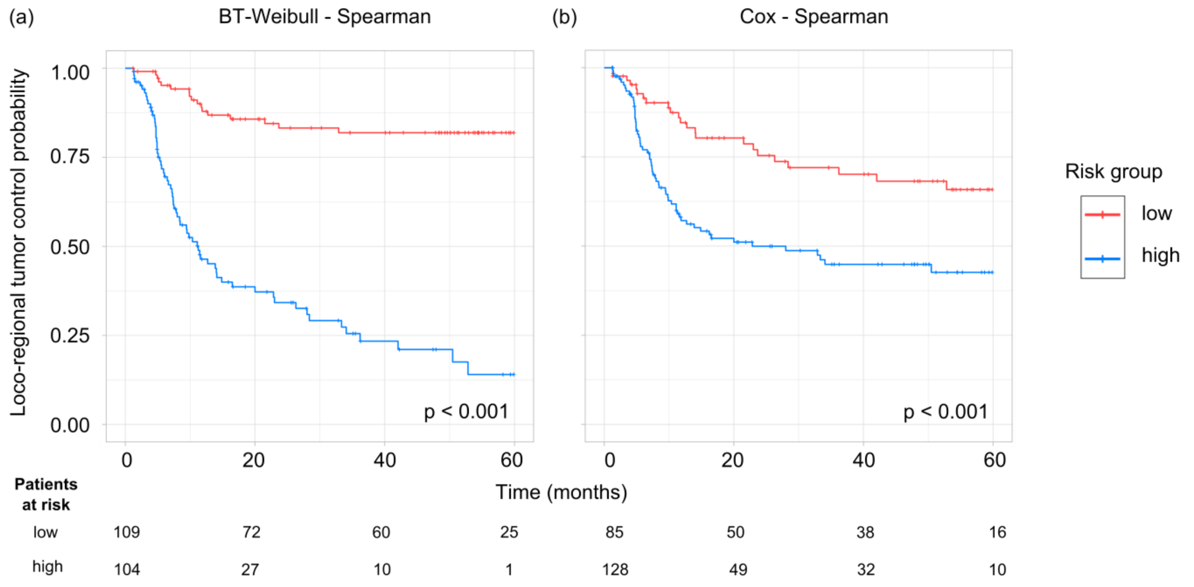

Supplementary Figure S7: Kaplan-Meier estimates of loco-regional tumour control for patients of the exploratory cohort stratified into a low and a high risk group. Both (a) the BT-

Weibull model in combination with Spearman feature selection and (b) the Cox model in combination with Spearman feature selection showed a significant patient stratification.

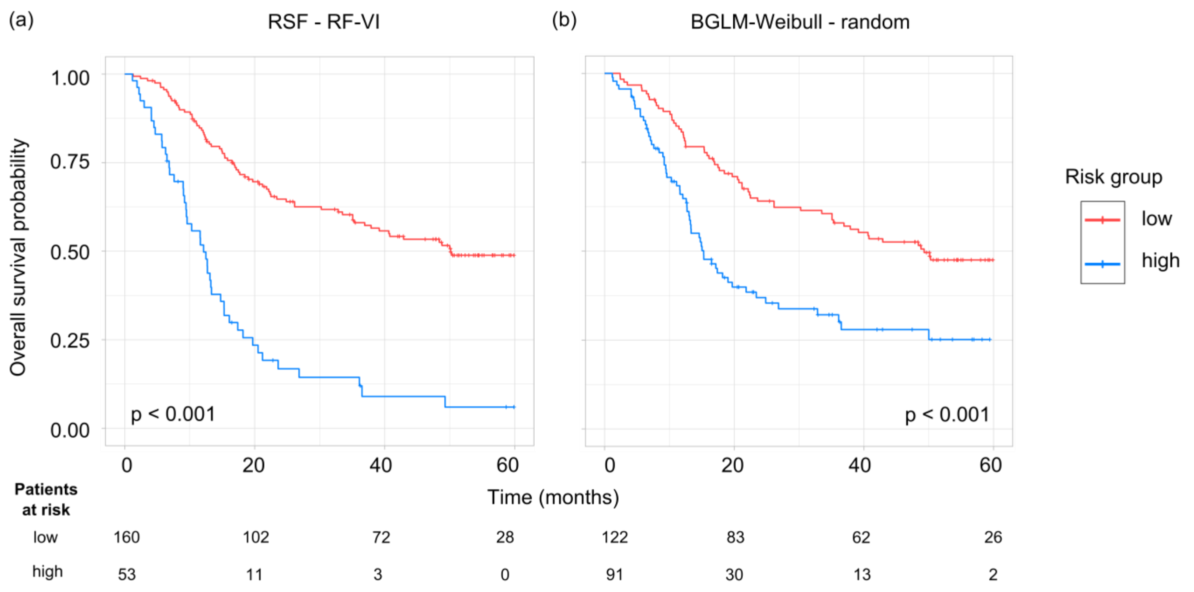

Supplementary Figure S8: Kaplan-Meier estimates of overall survival for patients of the exploratory cohort stratified into a low and a high risk group. Both (a) the RSF model in combination with RF-VI feature selection and (b) the BGLM-Weibull model in combination with random feature selection showed a significant patient stratification.

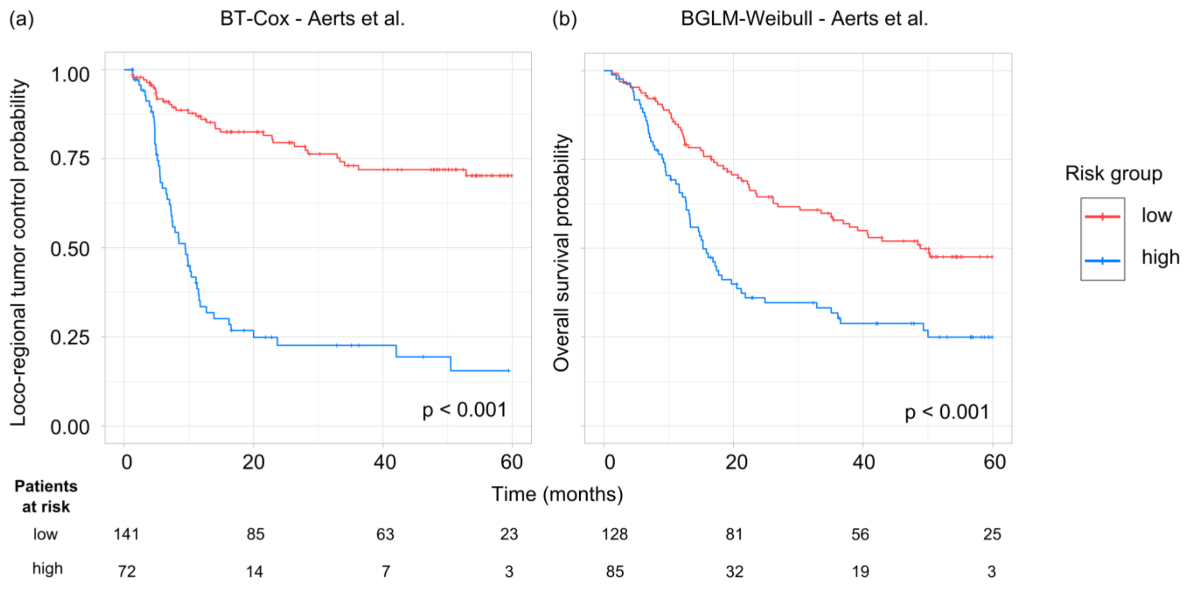

Supplementary Figure S9: Kaplan-Meier estimates for (a) loco-regional tumour control and (b) overall survival for patients of the exploratory cohort stratified into a low and a high risk

group. The signature by Aerts *et al.* [21] in combination with the BT-Cox and the BGLM-Weibull model showed a significant patient stratification.

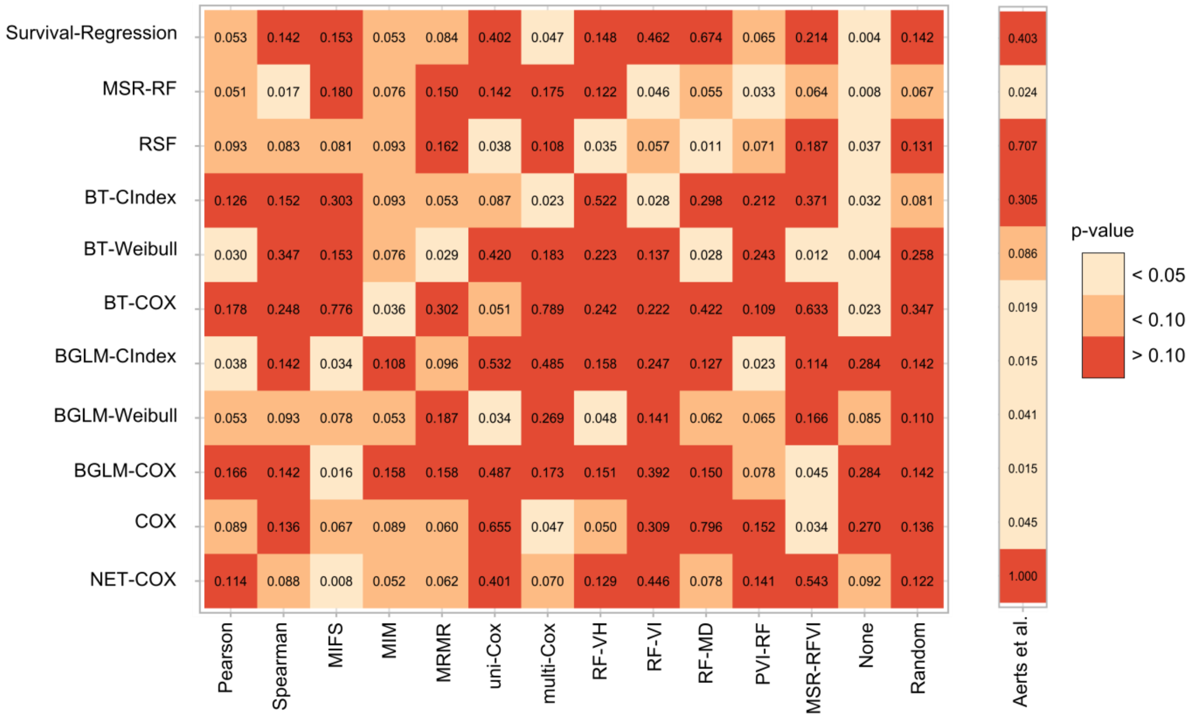

Supplementary Figure S10: *p*-values of the log-rank test for the considered feature selection methods and learning algorithms as well as the signature by *Aerts et al*. [21] for loco-regional tumour control. The cut-off values used for stratification were selected by 1000 bootstrap samples based on the exploratory cohort. The fraction of significant stratification results was calculated for each cut-off, leading to the optimal value which has the largest power. Cut-off values calculated on the exploratory cohort were applied to the validation cohort unchanged.

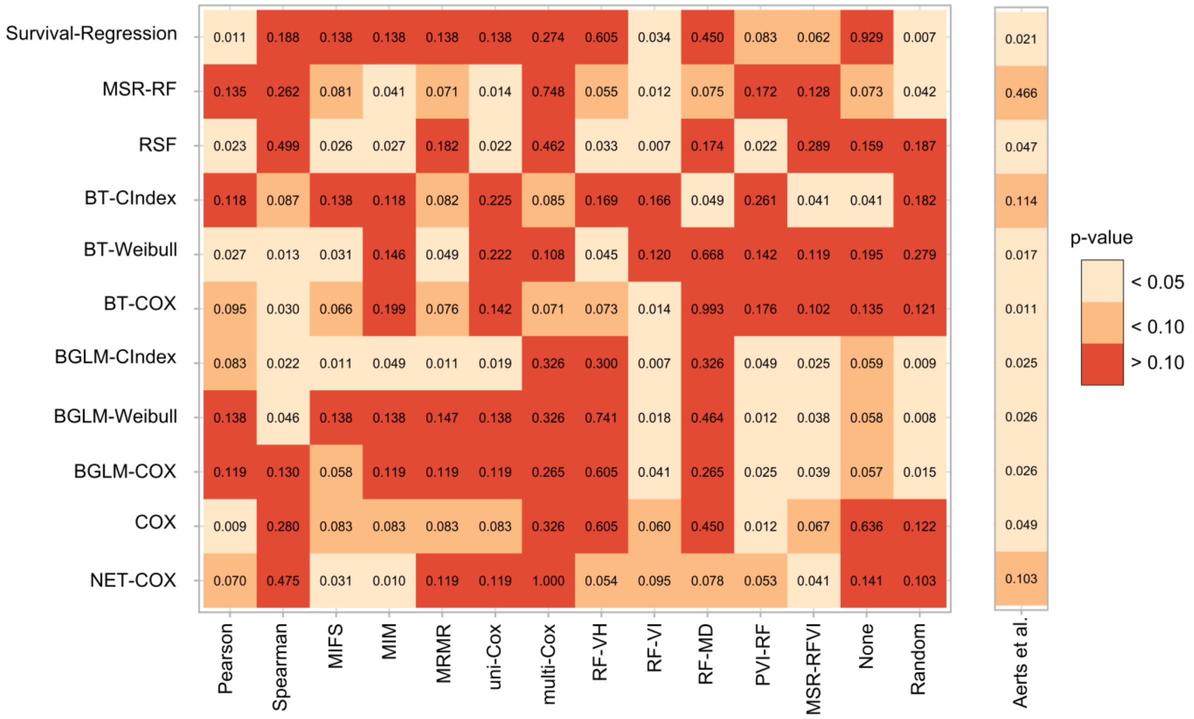

Supplementary Figure S11: *p*-values of the log-rank test for the considered feature selection methods and learning algorithms as well as the signature by *Aerts et al*. [21] for overall survival. The cut-off values used for stratification were selected by 1000 bootstrap samples based on the exploratory cohort. The fraction of significant stratification results was calculated for each cut-off, leading to the optimal value which has the largest power. Cut-off values calculated on the exploratory cohort were applied to the validation cohort unchanged.

References

[1] Rodgers JL, Nicewander WA. Thirteen Ways to Look at the Correlation Coefficient. Am Stat 1988;42:59. doi:10.2307/2685263.

[2] SPEARMAN C. Correlation culculated from faulty data. Br J Psychol 1910;3:271–95. doi:10.1111/j.2044-8295.1910.tb00206.x.

[3] Harrell FE, Lee KL, Mark DB. Multivariable prognostic models: Issues in developing models, evaluating asumptions and adequacy, and measuring and reducing error. Stat Med 1996;15:361–87. doi:10.1002/(SICI)1097-0258(19960229)15:4<361::AID-SIM168>3.0.CO;2-4.

[4] Penciana MJ, D'Agostino RB. Overall C as a measure of discrimination in survival analysis: Model specific population value and confidence interval estimation. Stat Med 2004;23:2109–23. doi:10.1002/sim.1802.

[5] Battiti R. Using Mutual Information for Selecting Features in Supervised Neural-Net

Learning. Ieee Trans Neural Networks 1994;5:537–50. doi:10.1109/72.298224.
[6] Peng H, Long F, Ding C. Feature selection based on mutual information: Criteria of Max-Dependency, Max-Relevance, and Min-Redundancy. IEEE Trans Pattern Anal Mach Intell 2005;27:1226–38. doi:10.1109/TPAMI.2005.159.
[7] Therneau T. Survival Analysis. CRAN 2016. doi:10.1007/978-1-4419-6646-9.
[8] Ishwaran H, Kogalur UB, Gorodeski EZ, Minn AJ, Lauer MS. High-Dimensional Variable Selection for Survival Data. J Am Stat Assoc 2010;105:205–17. doi:10.1198/jasa.2009.tm08622.
[9] Ishwaran H, Kogalur UB, Chen X, Minn AJ. Random survival forests for high-dimensional data. Stat Anal Data Min 2011;4:115–32. doi:10.1002/sam.10103.
[10] Ishwaran H, Kogalur UB. Random Forests for Survival, Regression and Classification (RF-SRC), R package version 1.6 2014.
[11] Wright MN, Dankowski T, Ziegler A. Random forests for survival analysis using maximally selected rank statistics. arXiv.org 2016:1–15.
[12] Wright MN, Ziegler A. ranger: A Fast Implementation of Random Forests for High Dimensional Data in C++ and R. arXiv Prepr 2015.
[13] Janitza S, Celik E, Boulesteix A-L. A computationally fast variable importance test for random forests for high-dimensional data 2015.
[14] Cox DR. Regression Models and Life-Tables. J R Stat Soc Ser B 1972;34:187–220. doi:10.2307/2985181.
[15] Simon N, Friedman J, Hastie T, Tibshirani R. Regularization paths for Cox's proportional hazards model via coordinate descent. J Stat Softw 2011;39:1–13. doi:10.18637/jss.v039.i05.
[16] Friedman J, Hastie T, Simon N, Tibshirani R. Package "glmnet": Lasso and Elastic-Net Regularized General Linear Models 2016:23.
[17] Package "mboost" Title Model-Based Boosting 2016.
[18] Ishwaran H, Kogalur UB, Blackstone EH, Lauer MS. Random survival forests. Ann Appl Stat 2008;2:841–60. doi:10.1214/08-AOAS169.
[19] Breiman L. Randomforest2001 2001:1–33. doi:10.1017/CBO9781107415324.004.
[20] Hosmer DW, Lemeshow S, May S. Applied Survival Analysis. vol. 41. Hoboken, NJ, USA: John Wiley & Sons, Inc.; 2008. doi:10.1002/9780470258019.
[21] Aerts HJWL, Velazquez ER, Leijenaar RTH, Parmar C, Grossmann P, Cavalho S, et al. Decoding tumour phenotype by noninvasive imaging using a quantitative radiomics approach. Nat Commun 2014;5. doi:10.1038/ncomms5006.
